# Adaptive Peptide Molecule as the Promising Highly-Efficient Gas-Sensor Material: In Silico Study

**DOI:** 10.3390/s23135780

**Published:** 2023-06-21

**Authors:** Alexander A. Petrunin, Maxim K. Rabchinskii, Victor V. Sysoev, Olga E. Glukhova

**Affiliations:** 1 Institute of Physics, Saratov State University, Astrakhanskaya Street 83, 410012 Saratov, Russia; sacha.petrynin@gmail.com; 2 Ioffe Institute, Politekhnicheskaya Street 26, 194021 Saint Petersburg, Russia; rabchinskii@mail.ioffe.ru; 3 Department of Physics, Yuri Gagarin State Technical University of Saratov, Polytechnicheskaya Street 77, 410054 Saratov, Russia; 4 Laboratory of Biomedical Nanotechnology, I.M. Sechenov First Moscow State Medical University, Trubetskaya Street 8-2, 119991 Moscow, Russia

**Keywords:** peptide, analyte, gas sensors, DFTB method, binding energy, electron density, adsorption center, local minimum of energy

## Abstract

Gas sensors are currently employed in various applications in fields such as medicine, ecology, and food processing, and serve as monitoring tools for the protection of human health, safety, and quality of life. Herein, we discuss a promising direction in the research and development of gas sensors based on peptides—biomolecules with high selectivity and sensitivity to various gases. Thanks to the technique developed in this work, which uses a framework based on the density-functional tight-binding theory (DFTB), the most probable adsorption centers were identified and used to describe the interaction of some analyte molecules with peptides. The DFTB method revealed that the physical adsorption of acetone, ammonium, benzene, ethanol, hexane, methanol, toluene, and trinitrotoluene had a binding energy in the range from −0.28 eV to −1.46 eV. It was found that peptides may adapt to the approaching analyte by changing their volume up to a maximum value of approx. 13%, in order to confine electron clouds around the adsorbed molecule. Based on the results obtained, the prospects for using the proposed peptide configurations in gas sensor devices are good.

## 1. Introduction

The Internet of Things (IoT) is the current mainstream trend in technology encouraging minds of the scientific and engineering community to leverage new data to digitalize manufacturing industry operations, evaluate human environments, and inspire new business models [1,2]. The expansion of IoT has advanced technologies for big-data capture, transfer, and analysis, such as machine learning [3], to collect useful information from the digital dust. Furthermore, the advancement of IoT has also given rise to an increase in the development of gas- and biosensing devices, by setting more stringent requirements on their functional parameters—sensitivity, selectivity, response/recovery time, and power consumption [4,5,6]. 

In this regard, chemiresistive sensors based on carbon nanomaterials, namely carbon nanotubes (CNTs) and graphene derivatives, have received considerable attention owing to their exceptional sensitivity, long-term stability, portability, and their independence from heat for operation [7,8,9]. The latter feature distinguishes sensing platforms based on carbon nanomaterials from the metal-oxide semiconducting chemiresistive gas sensors and optical sensors, which results in a drastic reduction in associated power consumption. However, the fundamental bottleneck of the CNT/graphene-based sensors is their poor selectivity, demonstrating a chemiresistive response to nearly all organic vapors and gases, while hindering the selective discrimination of a certain analyte [10]. 

Several strategies have been considered to overcome this issue. There are extensive applications of structural patterning and derivatization of carbon nanomaterials by certain functional groups to increase their affinity to a desired group of gases or volatile organic compounds (VOCs) over others [8,11,12]. Another approach to achieve distinctive detection of analytes is to apply such sets of derivatized carbon nanomaterials to fabricate a cross-reactive chemical sensor array or an on-chip multisensor array, forming biomimetic electronic noses (e-noses) [13,14,15]. In this context, the partially selective response of each sensor element contributes to the multiplexed response of the array. Further processing via pattern recognition systems involving machine learning algorithms allows us to identify and classify not only gases and VOCs but also complex aromas [16]. 

Despite the substantial increase in the selectivity of carbon-based sensors, these approaches retain some level of ambiguity in the identification of analytes, which is unsatisfactory for certain practical applications, such as the detection of explosives, viral and protein markers of certain diseases, and vital products of microorganisms [17,18]. Therefore, the aforementioned strategies should be complemented with further modification of a sensing layer of gas- or bio-recognizing biomolecules, such as aptamers and DNA strands, antibodies, proteins, mimicking olfactory receptors, and peptides [19,20,21,22,23]. Providing specific adsorption sites for a certain VOC or gas molecule, stemming from the unique pattern of multivalent interaction or complementary binding, these biomolecules can increase the sensitivity and selectivity of the developed sensors and E-nose units [23,24].

Among the tested biomolecules, short peptide sequences have been considered the most facile ones. Peptides combine high selectivity and fast response time, and can be easily designed using a virtual screening to bind chosen molecular targets. Furthermore, compared to antibodies or DNA strands, peptides are easily synthesized, purified, and immobilized on CNTs/graphene layers, thus, not substantially complicating the fabrication of sensors [22,25,26]. Given these advantageous properties, a set of sensing devices based on carbon nanomaterials with immobilized peptides has been fabricated and trialed. Cui et al. have reported on the development of an ultrasensitive trinitrotoluene (TNT) sensor based on graphene covered by GBP4 12-mer sequences [27]. Conversely, Lee et al. have designed and fabricated a sensor based on reduced graphene oxide (rGO), grafted with dinitrotoluene-specific binding peptide, and they could observe accurate discrimination of dinitrotoluene, a byproduct of TNT, with the limit of detection (LoD) below 80 ppm [23]. Furthermore, selective detection of odor molecules characterizing plant flavors, such as limonene, methyl salicylate, and menthol, has been achieved by graphene field-effect transistors with a self-assembled layer of three different peptides, against individual species of the target molecules [28]. Larisika M. et al. have demonstrated the fabrication of an olfactory biosensor based on a rGO field-effect transistor (FET), grafted by the odorant-binding protein14 (OBP14), to selectively detect odorants known to be attractive for bees, such as methylvanillate [29]. 

Despite these achievements, building a comprehensive library of gas- and bio-recognizing peptides is yet to be started. The current nomenclature of the applied peptides is far from complete or sufficient for current tasks in the selective detection of gases, VOCs, odors, and biomolecules. Thorough examinations of already-known prospective peptides, in terms of their conformation and affinity for adsorption to the complete set of desired analytes, the peculiarities in the charge transport between peptides and adsorbed analytes, and the LoD and response time of the designed sensor, are still to be performed. 

One of the powerful ways to solve these problems is a theoretical study using the methods of density functional theory (DFT) and density functional theory in the tight-coupling approximation (DFTB). As an example, the potential and mechanism of inhibition of corrosion of iron, copper, and aluminum by small peptides of aliphatic amino acids were studied in [30] utilizing the DFT and Monte Carlo computational methods. The authors calculated the adsorption energies and determined the most stable low-energy configurations for the adsorption of alanine amino acids and small peptides on Fe (110), Cu (111), and Al (111) surfaces. An empowering of the inhibitory effect by di- and tripeptides due to an increasing number of reaction centers of the molecular structure has been established. In another work of [31], it was shown that experimental methods do not always allow one to analyze the protein crown in situ. As a result, little is known about the effect of nanoparticles on the loosely bound proteins that form the soft crown. Nevertheless, it has been established by molecular dynamics and DFTB methods that hemoglobin can form either a hard or soft crown on silica nanoparticles, depending on pH conditions.

The purpose of this work is to identify the most sensitive peptide to a number of analytes, employing a computer simulation for further use in gas sensors. Five various peptides have been considered: CIHNP, CRQVF, DNPIQAVP, DSWAADIP, and WHVSC. Analyte molecules selected were alcohol molecules (methanol and ethanol), acetone, ammonia, benzene, hexane, trinitrotoluene, and toluene, which are of interest for their potential use in sensor detection.

## 2. Materials and Methods

### 2.1. Energy Calculation Details

Analyte adsorption on peptides was studied using the DFTB+, version 20.2 software package [32] using the density functional method in the tight-coupling approximation with a charge self-consistency (SCC-DFTB) [33,34] under energy expansion up to the third order (DFTB3) to significantly improve the description of charged systems and, particularly, the energy of hydrogen binding. The last opportunity yields options to significantly advance the accuracy in the description of organic molecules [35]. The Hubbard orbital correction for H, C, N, O, and S atoms was also applied. Within this method, the total energy of the system is calculated as

(1) $$E_{tot}=E^{H_{0}}+E^{\gamma }+E^{\Gamma }+E^{rep}$$

where $E^{H_{0}}$ is the energy of the band structure, $E^{rep}$ is repulsion energy, $E^{\gamma }$ is self-consistent charge energy, and $E^{\Gamma }$ is the third-order term of the Taylor series expansion of the exchange correlation energy. To describe the exchange-correlation interaction, the 3ob-3-1 parametrization was considered, which has proven itself in modeling organic molecules [36]. To correctly estimate the long-range interaction of analyte molecules with peptides, we used the Grimme D4 dispersion (DFTB + D4) [37,38]. The dynamic polarizabilities are preliminarily calculated by the time-dependent DFT to cover all the atomic elements up to radon (Z = 86) [39]. At the same time, applying Grimme correction allows us to derive results that match the ones acquired from the DFT method [40]. The electronic temperature was 300 K. In all calculations, the structures reached the energy minimum by the LBFGS method [41], with an accuracy of $10^{-4}$ eV/atom. Upon searching for an equilibrium atomic configuration, the analyte molecules turned and shifted relative to the peptides while finding the optimal position. This approach is one of the state-of-art and rapidly developing methods that provides physically correct results in the study of a large class of molecular and cluster structures. In particular, it was recently demonstrated [42] that the DFTB method solves the problem of finding the global minimum of the structure. For further instance, it was shown [43] that the DFTB approach, implemented in the DFTB+ software, provides a search for the global energy minimum in various systems including those based on several types of bonds, such as alkali, metal, and covalent clusters. The geometric volume of the peptide molecule, $V_{p}$, was calculated according to

(2) $$V_{p}=\left(x_{max}-x_{min}\right)\left(y_{max}-y_{min}\right)\left(z_{max}-z_{min}\right),$$

where $x_{max},\;y_{max},\;z_{max}$ are the maximum coordinates and $x_{min},\;y_{min},\;z_{min}$ are minimum coordinates along the x, y and z axes, respectively.

To quantitatively describe the interaction between peptides and analytes, the value of the binding energy is used, which most accurately characterizes the intensity of the interaction of the structure under study with the analyte. In this work, similar to others, the binding energy is calculated as the difference between the total energy of a final structure, $E_{peptide+analyte}$, and energies of isolated peptide structure, $E_{peptide}$, and analyte, $E_{analyte}$, according to

(3) $$E_{bind}=E_{peptide+analyte}-\left(E_{peptide}+E_{analyte}\right)$$

### 2.2. The Algorithm for a Search of Active Adsorption Centers

When describing an analyte adsorption from a fundamental viewpoint, the algorithm was developed for an analyte molecule approaching the peptides as follows.

1. Identification of the local adsorption centers. At this step, we develop a quantitative task to clarify the number of adsorption centers available. For this purpose, the electronic structure of all peptides under study is defined using an estimated distribution of electron density over the atoms of the structure. An analysis of the electron density map revealed several distinct local centers, accounting for the values of the excess electron charge. Thus, local centers with electronic charges ranging from −0.5e to −1.0e were identified. The charge range, of course, is rather conditional because it is individual for each structure. The local centers appear as confined 3D areas, including a group of atoms from one to three, characterized by the indicated excess electronic charge. Naturally, the local centers are primarily the atoms and groups of atoms able to actively interact with hydrogen atoms as a part of the analyte molecules.

2. Identification of the most active adsorption centers, from the standpoint of the binding energy characterizing the analyte molecules.

## 3. Results and Discussion

**Figure 1:**
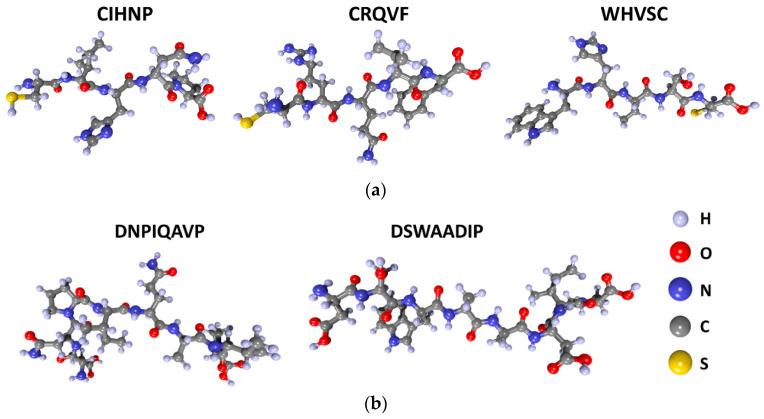
The atomistic structures of peptides: (**a**) CIHNP, CRQVF, and WHVSC; (**b**) DNPIQAVP and DSWAADIP.

Initial atomistic models of peptides were derived employing a tool for generating all the possible peptide combinations [44]. Further, they were optimized in order to obtain an equilibrium atomic configuration. Figure 1 yields the atomistic equilibrium structures for CIHNP, CRQVF, WHVSC (Figure 1a) and DNPIQAVP and DSWAADIP (Figure 1b) peptide molecules. As can be seen from the figure, the structures of all peptides are quite complex and developed. Therefore, the primary question is to identify the exact center for incoming analyte molecules, which is solved via examination of each peptide’s energy minimum. 

It is not possible to predict in advance such a binding site because all peptides contain a number of oxygen atoms which could serve as potential adsorption centers. For this challenge, an algorithm was applied to properly identify such centers.

### Search for Active Adsorption Centers

According to the first step of the algorithm for searching active adsorption centers, the electron density distributions were calculated for all the peptides under study by identifying local adsorption centers. Here, it is worth noting that the number of adsorption centers was chosen equal to eight for all the peptides because the molecular structures of peptides have approximately the same number of atoms and a similar overall structure.

**Figure 2:**
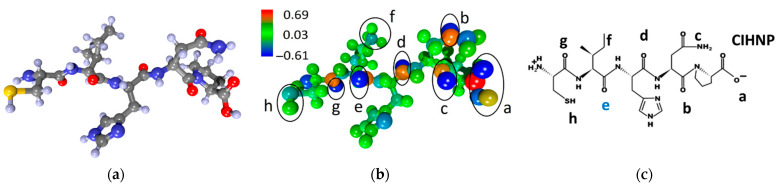
CIHNP peptide: (**a**) atomistic structure; (**b**) the distribution of electron density over atoms in values of electron charges; (**c**) the chemical formula. Letters of “a–h” mark the local adsorption centers for analytes. The letter “e” highlighted in blue indicates the most energetically active local center of this peptide.

Figure 2 presents the data on the CIHNP molecule: atomistic structure (Figure 2a), the electron charge density distribution over the atoms (Figure 2b), and the chemical formula (Figure 2c). The color scale shows an excess/deficiency of electronic charge when compared with the valency of a given atom. The charge calculation has been performed according to Mulliken’s population analysis. The red color corresponds to a lack of electronic charge, so the number is provided with a positive sign. The blue color corresponds to an excess of electronic charge so blue atoms are characterized by a negative charge. Figure 2b highlights eight local adsorption centers to be marked with (“a–h”) letters which are identified in the chemical formula (Figure 2c).

Let us consider in more detail the adsorption centers given in Figure 2b,c accounting for an excessive electron density. The center “a” is characterized by a charge of −0.70e, while other ones yield less negative charges equal to −0.58e (“b”), −0.61e (“c”), −0.59e (“d”), −0.59e (“e”), −0.27e (“f”), −0.58e (“g”), and −0.1e (“h”). All centers have approximately the same excess electronic charge of ~0.6e within ±0.1e. The observed variations in charges among the local adsorption centers are, obviously, due to the atoms located near these centers. The local centers with oxygen atoms (“a”, “b”, “c”, “d”, “g”) have a more negative charge compared to ones that are free of oxygen (“f”, “h”). Hence, most of the adsorption centers belong to oxygen atoms, in agreement with expectations. 

**Figure 3:**
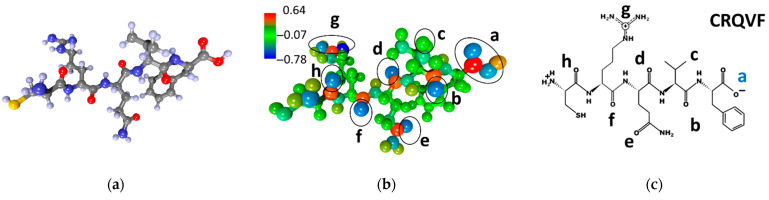
CRQVF peptide: (**a**) atomistic structure; (**b**) the distribution of electron density over atoms in terms of electron charges; (**c**) the chemical formula. Letters of “a–h” mark the local adsorption centers for analytes. The letter “a” highlighted in blue indicates the most energetically active local center of this peptide.

Similar searches for adsorption centers were carried out for all other peptides. Figure 3 shows the atomistic structure of the CRQVF peptide molecule (Figure 3a), the distribution of electron charge density over atoms (Figure 3b), and its chemical formula (Figure 3c). The detected local adsorption centers are also marked here, labeled with letters. The charging of the centers is distributed as follows: −0.41e (center “a”), −0.59e (center “b”), −0.56e (center “c”), −0.59e (center “d”), −0.58e (“e”—charge), −0.60e (center “f”), −0.36e (center “g”), −0.56e (center “h”).

These values are a bit lower but not significantly different than those observed in CIHNP centers; all the centers have approximately the same excess electronic charge of ~0.5e within ±0.1e. Again, the centers “b”, “d”, “e” and “f” have the largest excess charge. It is worth noting that, in this case, the S–H group of atoms does not act as an adsorption center, in contrast to the CIHNP peptide. In general, it can be assumed that just one of these centers of “b”, “d”, “e”, and “f” will act as the most energetically favorable and serve as a center for the adsorption of analyte molecules.

**Figure 4:**
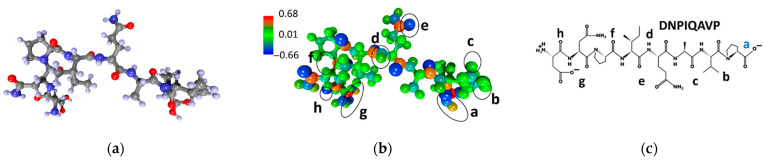
DNPIQAVP peptide: (**a**) atomistic structure; (**b**) the distribution of electron density over atoms in terms of electron charges; (**c**) the chemical formula. Letters of “a–h” mark the local adsorption centers for analytes. The letter “a” highlighted in blue indicates the most energetically active local center of this peptide.

The characteristics of the DNPIQAVP peptide molecule are shown in Figure 4 in the same arrangement as Figure 2 and Figure 3. According to the color scale of the electron charge density, it can be seen that adsorption centers can be located in local areas located directly adjacent to atoms or groups of atoms that carry an excess electronic charge. Figure 4a shows the atomistic structure, while Figure 4b is a map of the electron charge density distribution along with a charge scale. Here, the atomistic structure of the DNPIQAVP peptide lacks a sulfur atom that introduces certain changes in the pattern of the distribution of adsorption centers. The chemical formula of the DNPIQAVP peptide is given in Figure 4c. According to Figure 4, it can be seen that, in general, the scale of the electron charge density for the DNPIQAVP peptide coincides almost completely with the scale of the CIHNP peptide. In this regard, we can assume, furthermore, a similar arrangement of analyte molecules. For the DNPIQAVP peptide, adsorption center “a” is characterized by a charge of −0.39e, while centers of “b”, “c”, “d”, “e”, “f”, “g”, “h” have charges equal to −0.28e, −0.28e, −0.60e, −0.57e, −0.66e, −0.38e, −0.63e, respectively. In the case of this peptide, we again see that the local centers, which belong to oxygen atoms, the centers “f” and “h”, are the most electronegative ones when considering the Coulomb interaction.

**Figure 5:**
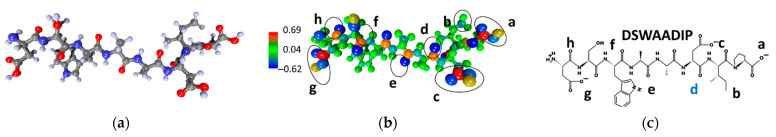
DSWAADIP peptide: (**a**) atomistic structure; (**b**) the distribution of electron density over atoms in terms of electron charges; (**c**) the chemical formula. Letters of “a–h” mark the local adsorption centers for analytes. The letter “d” highlighted in blue indicates the most energetically active local center of this peptide.

Figure 5 gives the details on the DSWAADIP peptide molecule. The structure of this peptide also lacks a sulfur atom, as does the DNPIQAVP peptide. The distribution of local adsorption centers is drawn in Figure 5b following its atomistic structure (Figure 5a). As can be seen from the figure, all centers are grouped into three and four atoms. The centers carry an excess charge as follows: “a”—−0.37e, “b”—−0.29e, “c”—−0.38e, “d”—−0.60e, “e”—−0.60e, “f”—−0.55e, “g”—−0.40e, “h”—−0.58e. In this case, one can see that the maximum excess charge does not exceed −0.60e, which appears at the centers of “d” and “e”.

**Figure 6:**
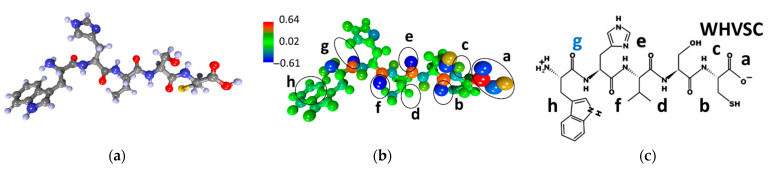
WHVSC peptide: (**a**) atomistic structure; (**b**) the distribution of electron density over atoms in terms of electron charges; (**c**) the chemical formula. Letters of “a–h” mark the local adsorption centers for analytes. The letter “g” highlighted in blue indicates the most energetically active local center of this peptide.

The last peptide molecule of WHVSC is shown in Figure 6. As in the previous cases, the atomistic structure (Figure 6a), the electron density distribution map (Figure 6b), and the chemical formula are present. This molecule contains a sulfur atom, but its presence does not affect the distribution of local adsorption centers, which are charged as follows (Figure 6b): −0.41e (“a”), −0.57e (“b”), −0.56e (“c”), −0.54e (“d”), −0.60e (“e”), −0.60e (“f”), −0.55e (“g”), and −0.47e (“h”). Similar to the DNPIQAVP peptide, the maximum excess charge does not exceed −0.60e. This suggests that, among all tested peptides, the peptides DNPIQAVP and DSWAADIP should exhibit the lowest binding energies to the analytes.

**Figure 7:**
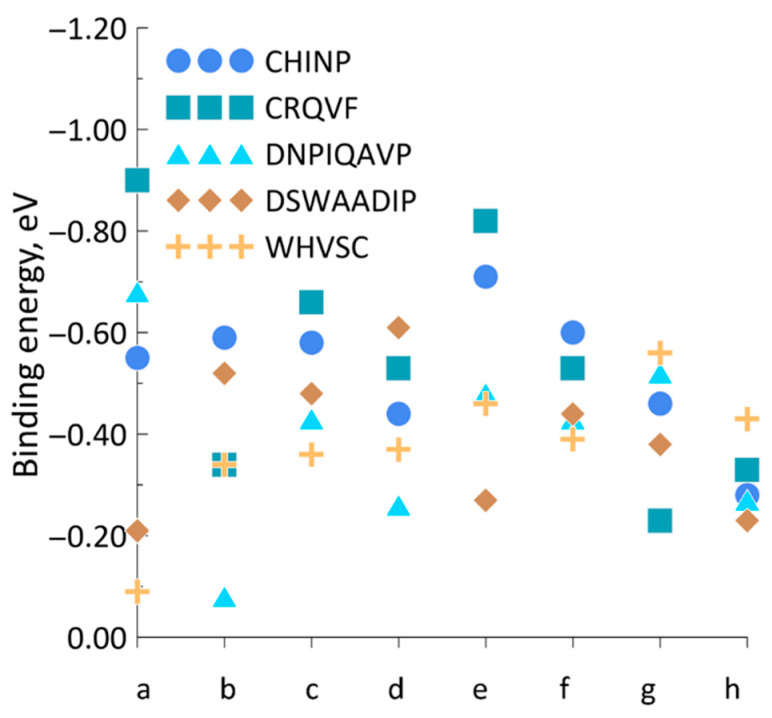
The values of the binding energy of the “peptide + acetone” systems, depending on acetone’s approaching a certain local center of “a–h” positions.

Further, according to the second step of the algorithm that searches for active adsorption centers, peptides were defined accounting for their binding energy with the analyte molecule. As a primary test analyte molecule, we chose acetone because it interacts very actively with organic structures. However, any of the analytes could be considered as a test molecule because the goal is to identify local centers with a “dense” electron density, which are shown for benzene and hexane molecules in Figure S1 (Supporting File). The acetone molecule was considered to be sequentially placed in all the local centers of “a”–“h” for each of the peptides. When approaching the acetone molecule to one of the local centers, optimization was carried out taking into account the van der Waals interaction; that is, we have varied all the coordinates of all atoms in order to find out a minimum of the total energy of the “peptide + analyte” system according to Equation (1). In each case, when the acetone molecule took an energetically favorable location at the local center following the optimization procedure, the binding energy was calculated in agreement with Equation (2). The results are given in Figure 7. Binding energy values are presented in the range from zero to the minimum value along the y-axis. This is done, for convenience, to determine the most preferred binding energy for acetone; the most favorable local minimum for each peptide is above all other points for this peptide. Based on the data in Figure 7, we may evaluate local centers by comparing their activities and choosing the most active adsorption center. Recall that in Figure 2, Figure 3, Figure 4, Figure 5 and Figure 6, the adsorption centers have a minimum binding energy marked in blue. As can be seen from Figure 7, the most active centers are: “e” with $E_{bind}$ = −0.71 eV (CIHNP), “a” with $E_{bind}$ = −0.90 eV (CRQVF), “a” with $E_{bind}$ = −0.68 eV (DNPIQAVP), “d” with $E_{bind}$ = −0.61 eV (DSWAADIP), and “g” with $E_{bind}$ = −0.56 eV (WHVSC).

**Figure 8:**
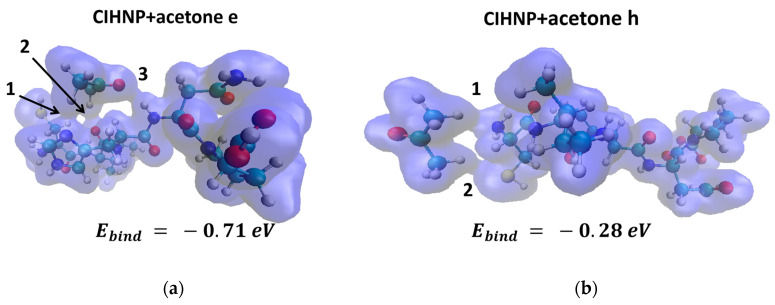
Electron density (isosurfaces of 0.015 atomic units) and structure of the CIHNP peptide when interacting with acetone molecule in the local adsorption centers with minimum (**a**) and maximum (**b**) binding energies. The numbers of 1, 2, 3 indicate the electron density contacts connecting the acetone molecule and the CIHNP peptide.

Thus, among all the peptides under study, CRQVF appears to be the most sensitive to acetone. The difference in binding energies for various local centers is due to the electron density in the area at the contact between acetone and peptides. Figure 8 shows the electron density, presented as iso-surfaces of 0.015 atomic units, in the general system of “CIHNP + acetone” in local centers with maximum binding energy (center “e”, Figure 8a) and minimum energy (center “h”, Figure 8b).

As one can see, in the case of the CIHNP + acetone (center “e”) system, the electron charge density forms three “legs” of electron density at the contact site, while in the case of CIHNP + acetone (center “h”) system, the electron density forms only two contacts. A more active interaction between the electron density of acetone and peptide in the case of CIHNP + acetone (center “e”) leads to a greater change in the binding energy and, accordingly, to more intense adsorption. As shown above, the local adsorption center “e” has a charge of −0.59e while the local center “h” has a charge of −0.1e. That is, a high charge in the local center prior to analyte appearance leads to a larger interaction between the electron density of the peptide and analyte molecules. According to [45], a positive charge might be localized near sulfur atoms. In our case, the S-containing peptides indeed have a region of positive charge near these atoms (Figure S2, Supporting File). The sulfur atoms of the CIHNP and CRQVF peptides have a charge of −0.25e; meanwhile, the charge of the sulfur atom in WHVSC is −0.20e, i.e., the total charge of these atoms remains negative. It does not affect the adsorption of the analytes since the interaction occurs between the electron density of analytes and peptides. Thus, after accounting for a charge, using Mulliken’s estimation method, the description of the physical processes in the structures under study was not erroneous.

**Figure 9:**
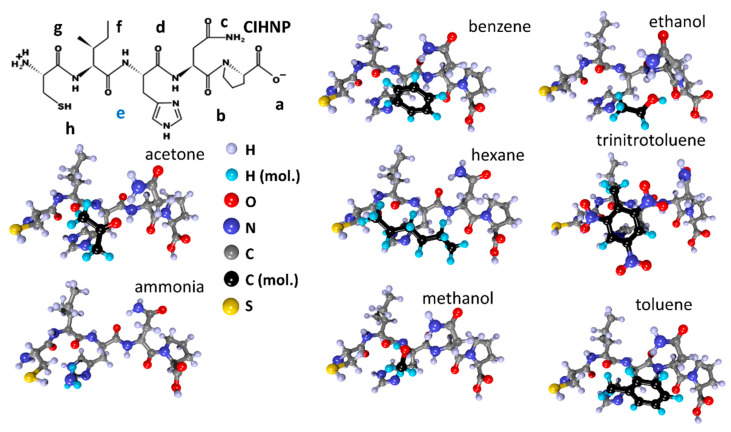
Structures of the CIHNP peptide after interacting with analyte molecules.

**Figure 10:**
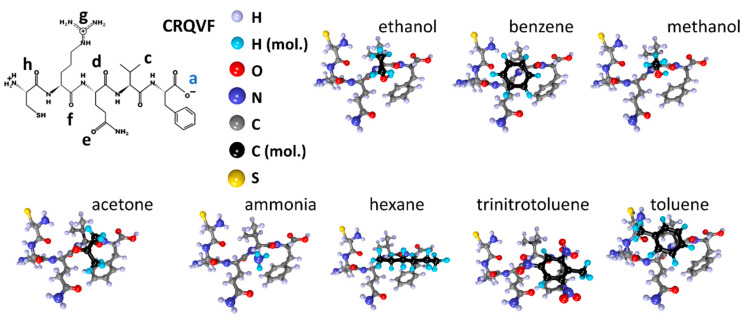
Structures of the CRQVF peptide after interacting with analyte molecules.

**Figure 11:**
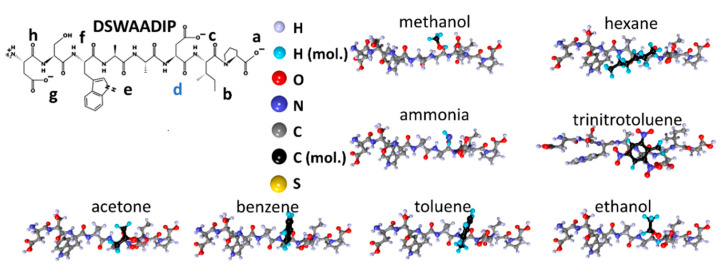
Structures of the DSWAADIP peptide after interacting with analyte molecules.

**Figure 12:**
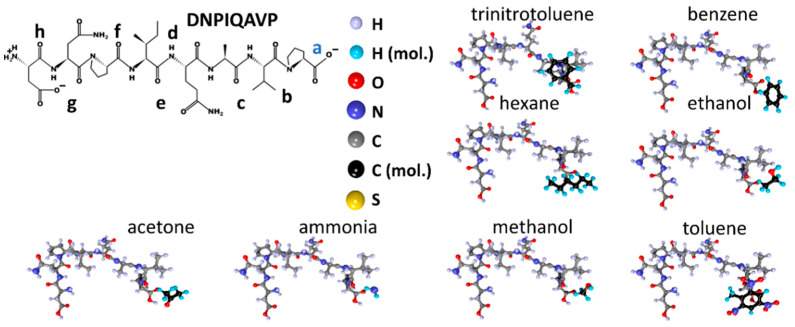
Structures of the DNPIQAVP peptide after interacting with analyte molecules.

**Figure 13:**
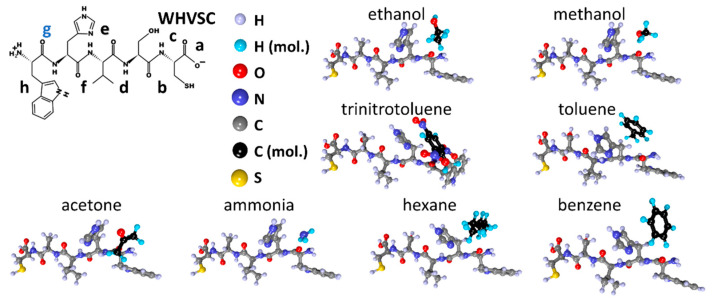
Structures of the WHVSC peptide after interacting with analyte molecules.

Next, molecules of the studied analytes were placed into the established local adsorption centers, with re-optimization of the “peptide + analyte” system. The structures for the CIHNP peptide are drawn in Figure 9 following an interaction with analytes. For visualization convenience, the carbon atoms of the analytes are highlighted in black, while the hydrogen atoms are colored turquoise blue. The structures of CRQVF + analyte, DSWAADIP + analyte, DNPIQAVP + analyte, and WHVSC + analyte are given in Figure 10, Figure 11, Figure 12 and Figure 13, respectively.

**Figure 14:**
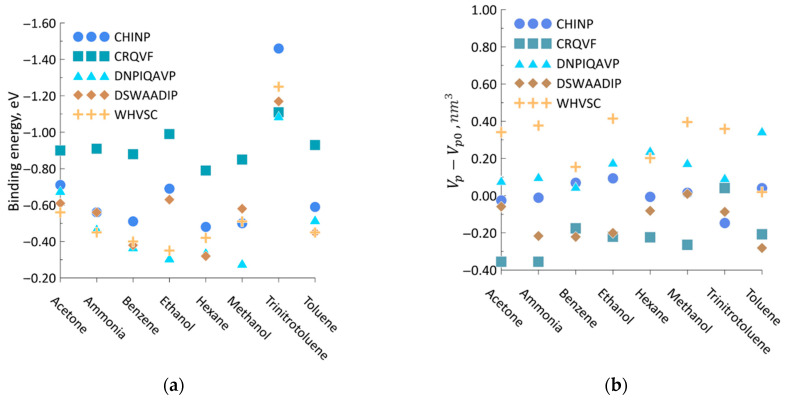
Characteristics of interaction between analytes and peptides: (**a**) binding energy; (**b**) change of peptide volume following an analyte approaching.

The binding energy is shown in Figure 14a as a result of the analyte approach. As can be seen from the figure, trinitrotoluene and ethanol molecules are the most active ones. At the same time, the hexane molecule exhibits the least activity. The maximum adsorption is observed for CRQVF (mean −0.8 eV) and CIHNP (mean −0.69 eV), while poorer adsorption is characteristic for DSWAADIP (mean −0.59 eV), WHVSC (mean −0.55 eV), and DNPIQAVP (mean −0.51 eV). This difference in adsorption for peptide/analyte configurations is associated with the structural features of both peptides and analytes.

The first remarkable structural feature of the peptide is the presence of sulfur atoms in three of the five peptides, namely CIHNP, CRQVF, and WHVSC. Sulfur atoms, as shown in [46,47], can significantly change the physical properties of materials and, in particular, improve the adsorption due to the presence of 2p and 3p valence orbitals [42]. The second structural feature is the existence of so-called electron “wells” which additionally promotes the adsorption of analytes. As shown in Figure 7, the bond between the analyte and peptide is strongest in a local energy minimum with a high electron density, which, in turn, is determined by the environment of atoms near this minimum. It can be seen from Figure 2, Figure 3, Figure 4, Figure 5 and Figure 6 that the global energy minimum is most often located in the places of accumulation of a large number of peptide atoms.

Also, the interaction of peptides with analytes is also significantly affected by the geometric adaptability of peptide molecules, i.e., a change in their size during the adsorption of analytes. Upon interaction with the analyte, the volume modification of peptides arises following a search for the energy minimum: the peptide re-arranges the atomic configuration in a manner to get the maximum electron density around the analyte. Figure 14b plots a change in peptide volume, $V_{p}$, following an interaction with various analytes. All the values are counted from the volume of the peptide, $V_{p0}$, observed prior to analyte approaching. Correspondingly, positive values of the volume change correspond to the expansion of peptides, while the negative values indicate their contraction. As can be seen from the figure, the CRQVF peptide, which has, on average, the maximum binding energy for all the analytes, is compressed most strongly when analytes are linked; the compression is about −0.35 nm^3^. The other peptides have, in general, similar trends, although they do not have a direct dependence on the peptide/analyte binding energy. Thus, the DSWAADIP peptide interacting with toluene is characterized by a maximum compression reaching −0.28 nm^3^. For CIHNP interacting with trinitrotoluene, this value is approx. −0.14 nm^3^. In contrast, compression no longer occurs for DNPIQAVP, suggesting that expansion of the peptide occurs when any analyte appears. Minimum expansion of DNPIQAVP is approx. 0.05 nm^3^, observed for benzene. The situation is similar for the WHVSC peptide, whose minimum expansion is ~0.02 nm^3^. In this case, the size of the adsorbed molecule plays a decisive role. In particular, upon adsorption of the largest molecule among considered ones, trinitrotoluene, the volume of peptides changes in the range from −0.15 nm^3^ to 0.36 nm^3^_,_ while for the smallest molecule of ammonium, volume changes from −0.35 nm^3^ to 0.38 nm^3^. The average values of peptide volume change are distributed as follows: CRQVF, −210.33 Å^3^; DSWAADIP, −141.82 Å^3^; CIHNP, 3.39 Å^3^; DNPIQAVP, 159.46 Å^3^; WHVSC, 282.97 Å^3^. As can be seen from these data, a change in the volume of the peptide does not lead to an unambiguous change in the binding energy, which is associated with the versatility of the physicochemical processes occurring during the interaction between analytes and peptides.

**Figure 15:**
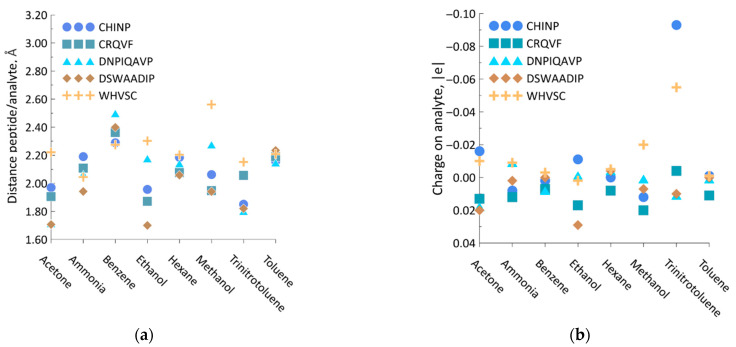
The characteristics of analyte/peptide interaction: (**a**) the minimum distance between the peptide and analyte upon interaction; (**b**) the charge on the analyte settling on peptide.

For all the cases of analyte insertion, the peptide/analyte distance was calculated. The data are shown in Figure 15a. It was found that the distance ranges from the smallest value of 1.700 Å, characterizing the DSWAADIP/ethanol system, up to the largest one of 2.562 Å, characterizing the WHVSC/methanol system.

In all cases, the minimum distance is between the hydrogen atom of the analyte molecule and the hydrogen atom of the peptide molecule. At the same time, nitrogen and oxygen atoms compose hydrogen bonds in the peptide/analyte interactions. The length of the N–H bond varies from 1.942 Å to 2.108 Å, while the O–H bond is within the 1.998–2.690 Å range. Therefore, the interaction of analytes with peptides does not result in appearing covalent bonds and the adsorption has a physical nature.

The nature of adsorption is naturally affected by the topology of the analyte molecule, namely, its geometric dimensions and mass. In our previous study on molecule adsorption over the surface of quasi-2D SnO_2_ [48], it was revealed that increasing the size of the molecule leads to stronger adsorption. However, this rule is not always fulfilled here due to the heterogeneity of local peptide adsorption centers. Thus, for the largest molecule, trinitrotoluene (227.13 g/mol), the most intense adsorption is, on average, −1.216 eV, while the average adsorption for the smallest ammonium molecule (18.039 g/mol) is less intense, −0.59 eV. At the same time, the hexane molecule, which is heavier than ammonium (86.178 g/mol), has the lowest binding energy, −0.47 eV on average, of all the cases under consideration.

In addition, the value of the re-distributed charge was calculated according to Mulliken for all the cases. The results are given in Figure 15b. Because the analyte molecule is located at a great distance from the peptide in all cases under study, and the formation of hydrogen bonds uses van der Waals forces, the value of the redistributed charge is in the range from ca. −0.100e to ca. +0.035e.

## 4. Conclusions

Among the result of studies using the DFTB3 + D4 method, features of interaction between CIHNP, CRQVF, WHVSC, DNPIQAVP, and DSWAADIP peptides with the analyte molecules of methanol, ethanol, acetone, ammonia, benzene, hexane, trinitrotoluene, and toluene were revealed. It was found out that, among the above peptides, CRQVF adsorbs analytes to the greatest degree, where the binding energy is ~1.57 higher, on average, than that of the most inactive peptide, DNPIQAVP. At the same time, the differences in adsorption for various peptide/analyte systems are associated with a number of reasons as follows.

1. The distribution of electron density around the peptide molecule is not homogeneous. For example, in the case of the CRQVF peptide, the binding energy can deviate from −57% to +66% of the average value (−0.54 eV) depending on the adsorption site.

2. The adaptability of peptide molecules, i.e., a decrease in their volume (maximum, by ca. 13%) creates a “denser” electron density around the adsorbed molecule.

3. The presence of sulfur atoms can improve the adsorption of analytes in the local minima of peptides, which is in accordance with data given in other works.

4. The topology of the adsorbed molecule itself can impact adsorption. For example, the binding energy of the peptide with the heaviest analyte molecule, TNT, is ~2.06 times greater than that of the lightest molecule, ammonia.

This work shows that the binding energy of the test analytes varies depending on the peptide, and was in the range of −12.91–−20.76 kcal/mol for acetone, −10.38–−20.99 kcal/mol for ammonia, −8.53–−20.29 kcal/mol for benzene, −7.15–−22.83 kcal/mol for ethanol, −7.38–−18.22 kcal/mol for hexane, −6.46–−19.60 kcal/mol for hexane, −25.14–−33.67 kcal/mol for trinitrotoluene, and −10.38–−21.45 kcal/mol for toluene. These values allow us to consider CIHNP, CRQVF, DNPIQAVP, DSWAADIP, and WHVSC peptides as promising materials for designing gas sensors. For comparison, in earlier studies [49], the PA-1 peptide complex exhibits a bond energy upon adsorption of benzene and toluene molecules to be −4.23 kcal/mol and −6.25 kcal/mol, respectively. In another work [24], the binding energy characterizing the OBPPs peptides is observed in the range of −2.82–−3.02 kcal/mol for acetone, −1.01–−1.74 kcal/mol for ammonia, −2.37–−2.55 kcal/mol for ethanol, and −3.57 to −4.47 kcal/mol for toluene.

Based on the simulation results obtained, it can be concluded that peptide molecules are promising to be applied in electronic gas sensors in order to detect organic molecules from the air.

## Data Availability

Not applicable.

## Supplementary Materials

The following supporting information can be downloaded at: https://www.mdpi.com/article/10.3390/s23135780/s1. Figure S1. The values of the binding energy of the CIHNP + acetone, CIHNP + benzene, CIHNP + hexane systems depending on acetone’s approaching a certain local center. Figure S2. The electron density of the CIHNP peptide. The areas of positive charge are marked in red, and the areas of negative charge are marked in blue. Isosurfaces of 0.01 atomic units.
