# Supplementary material for: Adaptive Peptide Molecule as the Promising Highly-Efficient Gas-Sensor Material: In Silico Study
Source: Sensors (Basel). 2023 Jun 21;23(13):5780. doi: 10.3390/s23135780 (PMC10346805; doi:10.3390/s23135780)
Supplement: Supplementary file 1 [file sensors-23-05780-s001.zip › sensors-2384714-supplementary.pdf]

Supporting Information

to

Adaptive Peptide Molecule as the Promising Highly-Efficient Gas-Sensor Material: In Silico Study

Alexander A. Petrunin [1], Maxim K. Rabchinskii [2], Victor V. Sysoev [3,*] and Olga E. Glukhova [1,*]

[1] Institute of Physics, Saratov State University, Astrakhanskaya Street 83, 410012 Saratov, Russia; sacha.petrynin@gmail.com
[2] Ioffe Institute, Politekhnicheskaya Street 26, 194021 Saint Petersburg, Russia; rabchinskii@mail.ioffe.ru
[3] Department of Physics, Yuri Gagarin State Technical University of Saratov, Polytechnicheskaya Street 77, 410054 Saratov, Russia
[4] Laboratory of Biomedical Nanotechnology, I.M. Sechenov First Moscow State Medical University, Trubetskaya Street 8-2, 119991 Moscow, Russia
* Correspondence: vsysoev@sstu.ru (V.V.S.); glukhovaoe@info.sgu.ru (O.E.G.); Tel.: +7-8452-514-562 (O.E.G.)

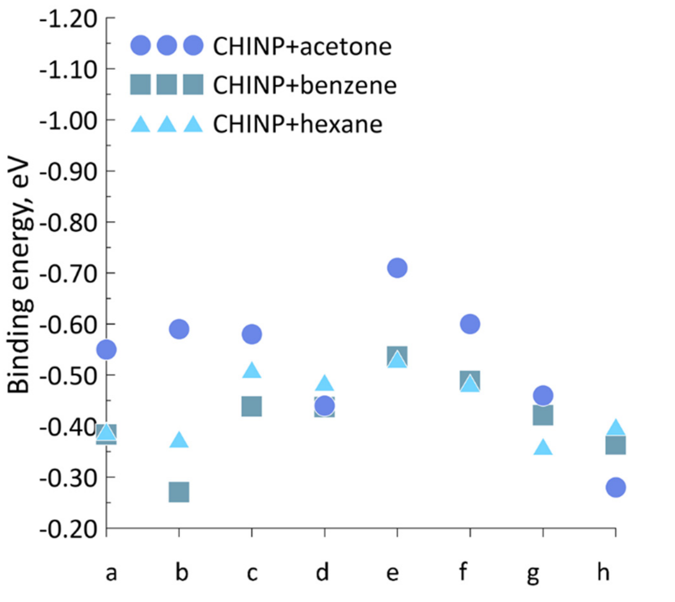

Figure S1. The values of the binding energy of the CIHNP+acetone, CIHNP+benzene, CIHNP+hexane systems depending on acetone's approaching a certain local center.

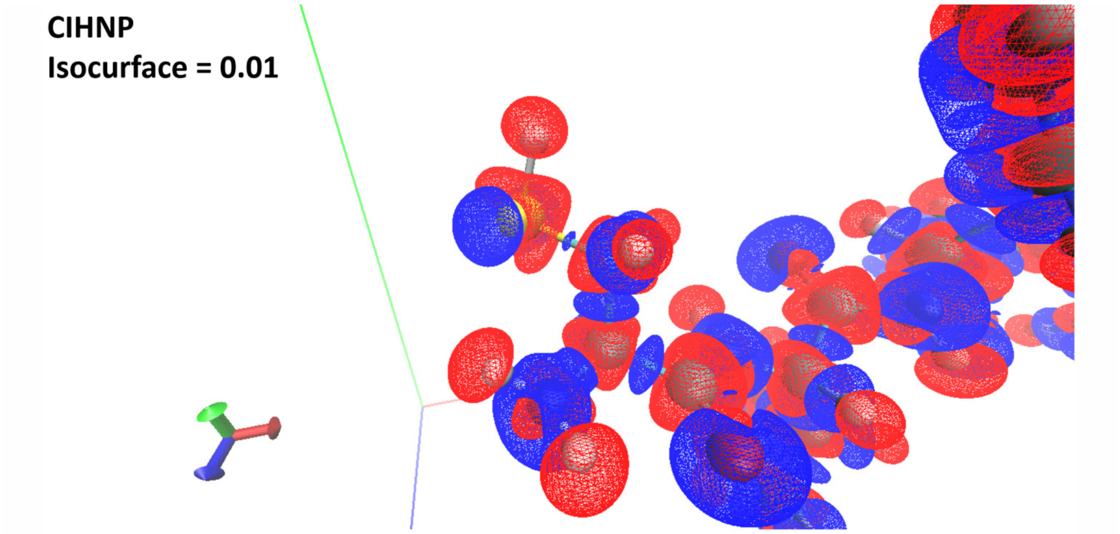

Figure S2. The electron density of the CIHNP peptide. The areas of positive charge are marked in red, and the areas of negative charge are marked in blue. Isosurfaces of 0.01 atomic units.
